# Genetic Insights into the Impact of Complement in Alzheimer’s Disease

**DOI:** 10.3390/genes12121990

**Published:** 2021-12-15

**Authors:** Megan Torvell, Sarah M. Carpanini, Nikoleta Daskoulidou, Robert A. J. Byrne, Rebecca Sims, B. Paul Morgan

**Affiliations:** 1 UK Dementia Research Institute Cardiff, School of Medicine, Cardiff University, Cardiff CF24 4HQ, UK; TorvellM@Cardiff.ac.uk (M.T.); CarpaniniS@Cardiff.ac.uk (S.M.C.); daskoulidoun1@cardiff.ac.uk (N.D.); ByrneR8@cardiff.ac.uk (R.A.J.B.); 2 Division of Infection and Immunity, Systems Immunity Research Institute, School of Medicine, Cardiff University, Cardiff CF14 4XN, UK; 3 Division of Psychological Medicine and Clinical Neuroscience, School of Medicine, Cardiff University, Cardiff CF24 4HQ, UK; SimsRC@cardiff.ac.uk

**Keywords:** complement, complement receptor 1, clusterin, late-onset Alzheimer’s disease, genetics, neuroinflammation

## Abstract

The presence of complement activation products at sites of pathology in post-mortem Alzheimer’s disease (AD) brains is well known. Recent evidence from genome-wide association studies (GWAS), combined with the demonstration that complement activation is pivotal in synapse loss in AD, strongly implicates complement in disease aetiology. Genetic variations in complement genes are widespread. While most variants individually have only minor effects on complement homeostasis, the combined effects of variants in multiple complement genes, referred to as the “complotype”, can have major effects. In some diseases, the complotype highlights specific parts of the complement pathway involved in disease, thereby pointing towards a mechanism; however, this is not the case with AD. Here we review the complement GWAS hits; *CR1* encoding complement receptor 1 (CR1), *CLU* encoding clusterin, and a suggestive association of *C1S* encoding the enzyme C1s, and discuss difficulties in attributing the AD association in these genes to complement function. A better understanding of complement genetics in AD might facilitate predictive genetic screening tests and enable the development of simple diagnostic tools and guide the future use of anti-complement drugs, of which several are currently in development for central nervous system disorders.

## 1. Alzheimer’s Disease, Inflammation, and Complement

Alzheimer’s disease (AD) is a common, chronic neurodegenerative disease. There are currently over 50 million cases of AD worldwide, and with an increasingly ageing population, this number will increase further [1]. AD is associated with a progressive decline in cognitive function and memory and a reduced ability to carry out day-to-day tasks, culminating in a complete loss of independence. Pathologically, AD is characterised by a build-up of protein deposits (amyloid-β (Aβ) plaques and hyperphosphorylated tau tangles) throughout the brain. Cognitive impairment is a consequence of regional neuronal and synapse loss. These events are accompanied by an inflammatory response: astrocytes and microglia, the innate immune cells of the brain, adopt a neurotoxic, phagocytic, proinflammatory phenotype and interact with plaques, tangles, and damaged or dying neurons [2]. It is increasingly apparent that the neuroinflammatory response is a driving force in AD pathology rather than a bystander or consequence of disease; perhaps the clearest evidence comes from genetic studies. Many of the genes most strongly associated with AD risk are involved in inflammation and immunity [3,4]. These data make it imperative to better understand when, where and how inflammation occurs in the course of AD in order to design better tests and novel drugs.

The complement system is an important component of the innate immune system and a potent driver of inflammation; it is the first line of defence against invading microorganisms and a key player in garbage disposal systems throughout the body. Through a tightly coordinated cascade of events, complement mediates pathogen recognition and destruction either via opsonisation followed by phagocytosis or by the formation of a lytic pore, the membrane attack complex (MAC). These processes are accompanied by the production of anaphylatoxins, C3a and C5a, which drive inflammation and facilitate immune cell recruitment.

**Figure 1:**
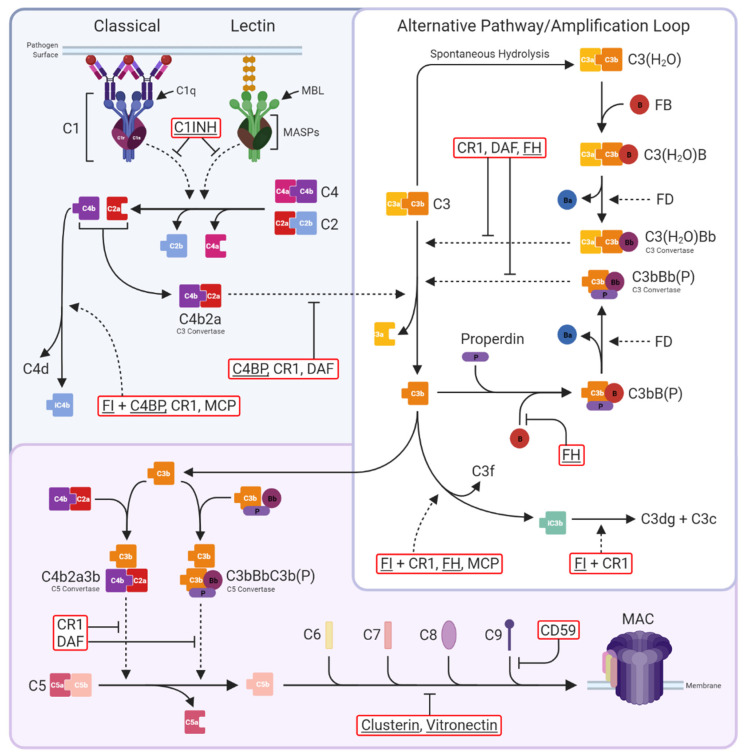
The complement system. Three activation pathways converge on the central C3 molecule. The classical pathway is triggered by binding of antibody-antigen complexes to C1 via C1q subunits. C1r proteolytically activates C1s, which in turn cleaves C4 and C2 to form the classical C3 convertase C4b2a. The lectin pathway begins with recognition of pathogen surface carbohydrates by mannose-binding lectin (MBL) followed by activation of MBL-associated serine proteases (MASPs), which also cleave C4 and C2 to generate C4b2a. The alternative pathway is an amplification loop initiated by C3b generated in the above activation pathways or by spontaneous hydrolysis of C3 to C3(H_2_O). Factor B (FB) then binds C3b/C3(H_2_O), enabling its cleavage by Factor D (FD) to form the alternative pathway C3 convertase C3bBb/C3(H_2_O)Bb; binding of properdin (P) stabilises the convertase. Both C3 convertases cleave C3 into C3a and C3b. The classical and lectin pathways are negatively regulated by C1-inhibitor (C1INH), which inhibits both C1s and MASPs, while the C3 convertases are regulated by C4b-binding protein (C4BP; specific for C4b2a), decay-accelerating factor (DAF; specific for C3bBb), complement receptor 1 (CR1), and Factor H (FH), either directly through increasing decay or indirectly by catalysing cleavage of C4b by Factor I (FI). At the next stage of the pathway, C3b is incorporated into the C3 convertases to form the C5 convertases C4b2a3b and C3bBbC3b(P). These are regulated in the same manner as the C3 convertases and cleave C5 into C5a and C5b to trigger the terminal pathway. C5b is sequentially bound by C6, C7, C8, and up to 18 C9 molecules to form the membrane attack complex (MAC); MAC assembly is inhibited by clusterin and vitronectin in the fluid phase and CD59 on cells. Complement regulators are in red boxes, fluid-phase regulators are underlined. Solid, dotted, and blunt arrows indicate pathway progression, proteolytic cleavage, and direct inhibition, respectively.

Complement can be activated through one of three pathways; classical (CP), lectin (LP), or alternative (AP) (Figure 1). CP activation is initiated by binding of the C1q/r/s complex to a surface either via surface-bound antibodies (IgG and IgM) or a variety of self-molecules such as Aβ and apoptotic markers including phosphatidylserine and extracellular DNA [5,6]; binding triggers activation of C1s, a serine protease that cleaves C4 and C2 to produce the membrane-bound C3-convertase, C4b2a. The LP is activated by mannose-binding lectin (MBL) or ficolin, these bind carbohydrate epitopes on surfaces. MBL-associated serine proteases (MASPs) cleave C4 and C2 to generate C4b2a, as in the CP. The AP is better considered as an amplification loop whereby either spontaneously hydrolysed C3 (C3(H_2_O)) or C3b generated in the CP/LP, bind factor B (FB), catalysing FB cleavage by factor D (FD) to form the AP C3-convertase (C3(H_2_O)Bb or C3bBb), which cleaves more C3 to generate membrane-bound opsonin C3b. The AP loop is therefore self-perpetuating and rapidly activating, critical for successful pathogen clearance, but dysregulation can be extremely costly. The three pathways converge at the point of C3 cleavage; each C3-convertase cleaves multiple C3 molecules into C3a and C3b leading to widespread complement deposition. C3b binding adjacent to C3-convertases creates the C5-convertases C4b2a3b and C3bBb3b, which cleave C5 into C5a and C5b. C3a and C5a are potent proinflammatory anaphylatoxins that recruit and activate immune cells expressing C3a and C5a receptors. C5b sequentially recruits C6, C7, C8, and C9 to form the membrane attack complex (MAC), which through a series of conformational changes, punches through the cell membrane resulting in cell lysis or cell activation.

To avoid damage to self, complement is tightly controlled at every level of the pathway by an array of regulators in fluids and on cell surfaces (Figure 1). Nevertheless, over-activation or failure of complement regulators to keep the pathway in check can trigger a vicious cycle of inflammation and tissue damage.

## 2. Genetics Implicate Inflammation, Immunity, and Complement in the Pathogenesis of Late-Onset AD

Late-onset AD (LOAD), responsible for ~95% of AD cases, is a multifactorial disease with a heritability of over 58% [7]. Since 2009, large genome-wide association studies (GWAS) have identified over 75 independent genetic risk factors for LOAD [3,8,9,10]. In silico pathway analyses have implicated amyloid and tau processing, lipid, and innate immunity pathways [4]. Approximately 20% of LOAD risk loci encode proteins implicated in immunity; many of these have roles in macrophage and microglial activation, an observation supported by recent single-cell expression enrichment analyses [8]. Among the GWAS statistically significant (GWS) hits are two genes encoding proteins of the complement pathway; *CR1* encoding the membrane protein complement receptor 1 (CR1) and *CLU* encoding the plasma regulator clusterin. Additionally, *C1S* encoding the enzyme C1s reaches near GWS in the most recent GWAS [8]. *CR1* and *CLU* are among the most significant GWAS hits, ranking high in the top 10. These strong associations provide the impetus for this review of complement genetics in LOAD.

## 3. Complement Genetic Variation Impacts Risk of Inflammatory Disease

Genetic variations within complement genes are extremely widespread in the general population; over the last 20 years, many common polymorphisms and rare mutations in complement genes have been linked with diverse inflammatory and infectious diseases, demonstrating the pivotal role of the complement pathway in determining disease risk (Table 1). Occasionally, these genetic variants are the primary cause of a disease through either causing deficiency or significant gain or loss of function changes in complement components or regulators; more commonly, functional changes associated with variants are subtle and exacerbate existing pathology by contributing to a vicious cycle of inflammation and tissue damage.

Considering the common polymorphisms, individual variants usually have only minor effects on protein function and complement homeostasis, but the additive effects of combinations of variants in multiple complement genes can have major effects, tipping the balance in favour of complement dysregulation and impacting disease predisposition. The combination of common genetic variants in complement genes that defines the complement genetic make-up of an individual is referred to as the “complotype” [11].

The complotype has been best studied in the context of age-related macular degeneration (AMD), progressive retinal disease, and the leading cause of blindness in the developed world. Common variants in genes encoding the AP components C3 and FB and the AP regulator FH are individually associated with higher C3 convertase activity and increased AMD risk; a combination of risk variants in these three genes (*C3* (rs2230199), *CFB* (rs641153), and *CFH* (rs800292)) increased complement activity in plasma six-fold [12]. This complotype, and another *CFB* variant (rs4151667), were later associated with AMD disease status and increased complement activation markers (C3d/C3 ratio) in AMD plasma [13]. These variants were also associated with an increased risk of dense deposit disease (DDD), a renal disease characterised by systemic AP activation and complement deposition in the kidneys. In contrast, the AP gene variants conferring risk for AMD and DDD were not risk variants for another renal disease associated with complement dysregulation, atypical haemolytic uremic syndrome (aHUS), a disease characterised by thrombocytopenia, microangiopathic haemolytic anaemia, and acute renal failure with complement deposition in the kidney [14]. This lack of concordance of risk suggests that the roles of complement are quite different in these superficially similar diseases; in support of this, a common genetic variation that causes deletion of the genes encoding FH-related proteins 1 and 3 (*CFHR1*/*CFHR3*) is protective for AMD but increases the risk of aHUS [14]. These findings demonstrate that the same complement gene variant, or set of variants, can be involved in several diseases and that specific variants may have inverse effects on risk in some apparently similar diseases. Better knowledge of the effects of these variants on complement regulation in plasma and in tissues will inform understanding of mechanisms of disease.

## 4. Complement in LOAD

**Figure 2:**
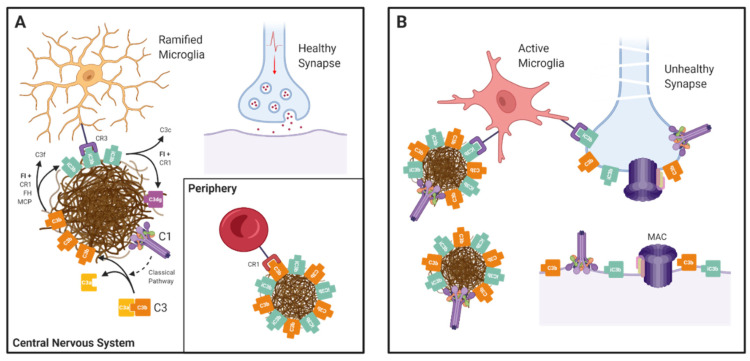
The Janus-faced nature of complement in AD: (**A**) In the central nervous system complement components and activation products (C1q/r/s and C3b) are deposited on amyloid plaques. C3b is converted to iC3b by Factor I (FI) with cofactor activity from CR1, Factor H, or MCP. iC3b binds to phagocytic receptor CR3 (an integrin dimer comprising CD11b and CD18 chains) on the surface of microglia, enabling plaque clearance. iC3b is further broken down by FI and CR1 into inactive C3dg. In the periphery, CR1 binds to C3b-opsonised amyloid aggregates and transports them to the liver for destruction in a process called “immune complex clearance”. (**B**) Complement dysregulation tips the balance towards destruction. In the absence of proper CR1 function, complement components accumulate, resulting in cell activation or damage. Complement is also involved in pathological synapse loss in AD. C1 binds to a poorly defined receptor on synapses and triggers classical pathway activation, resulting in C3b opsonisation and subsequent phagocytosis by activated microglia.

In post-mortem analyses of LOAD brain, complement components and activation products, notably C1q, C4b, C3b/iC3b, and MAC, co-localise with amyloid plaques and neurofibrillary tangles [5,15,16,17]. By default, these studies only address late/end-stage disease and provide no clues as to how complement activation impacts the disease. Given the role of complement in “taking out the trash”, one likely role of complement in LOAD is in facilitating the removal of accumulated amyloid plaques and tangles, dead and dying cells. Indeed, Aβ peptides, the precursors of amyloid, when exposed to serum, activate both the CP and AP and are opsonised by C3b/iC3b fragments [18]; this would enable recognition and phagocytosis by cells expressing complement receptor CR3, including CNS resident microglia (Figure 2). Outside of the brain, C3b-opsonised Aβ aggregates can bind CR1 on erythrocytes, a pathway for clearance in the liver [19]. These findings suggest that complement activation may have a protective role in early disease, provoking local phagocytosis of amyloid by resident cells and peripheral clearance; however, complement is a double-edged sword, protective when properly regulated but with the potential to cause damage when dysregulated. Dysregulated complement can then drive inflammation and directly activate or damage self-cells. Importantly, complement activation has been implicated in synapse pruning and loss, both physiological during brain development and pathological in neurodegeneration [20,21,22,23]. C1 tags synapses destined for removal and trigger CP activation leading to deposition of opsonic C3 fragments, signalling microglial phagocytosis. The demonstration that mice deficient in C1q or C3 show reduced synapse loss emphasises the importance of this process [23].

Whether complement activation is beneficial or detrimental for LOAD progression depends on regulation. Inappropriate activation or dysregulation of complement will drive pathological inflammation and has been implicated in inflammatory brain diseases such as neuromyelitis optica and multiple sclerosis [24,25]. The strongest evidence implicating complement in LOAD aetiology comes from genetic studies; genome-wide association studies (GWAS) implicated *CR1* and *CLU,* respectively encoding the complement receptor CR1 and the fluid-phase regulator clusterin [3,9,26]; the most recent LOAD GWAS reported a novel suggestive association of *C1S* the gene encoding the critical CP enzyme C1s, with risk [8]. Below we will briefly describe each of these complement hits, address the nature of their LOAD associations and explore mechanisms.

## 5. CR1

### 5.1. Function

CR1 is a receptor for the complement activation products C3b and C4b and a number of other ligands, detailed below. Once bound to CR1, C3b and C4b can be cleaved by the plasma protease FI, with CR1 itself providing the essential cofactor activity. The cleavage products (iC3b and C4c, respectively) have a minimal affinity for CR1; this binding-cleavage-release cycle is critical for the role of CR1 in immune complex (IC) handling [27]. C3b/C4b-coated ICs bind CR1 on erythrocytes in the circulation and are ferried to the liver and spleen for transfer to tissue macrophages expressing CR3 (the receptor for iC3b, now abundant on the IC) for phagocytic elimination. CR1 also has decay-accelerating activity for the C3 and C5 convertases; it binds C4b displacing C2a and binds C3b displacing Bb; this capacity to decay CP and AP convertases confers powerful complement regulating activity, although this is likely of minor physiological importance.

### 5.2. Expression

CR1 is expressed on erythrocytes where it performs the critical IC transport role described above; indeed, reduced CR1 levels on erythrocytes is strongly associated with the immune complex disease systemic lupus erythematosus (SLE), although whether this is cause or effect remains a subject of debate [28]. CR1 is also expressed on leukocytes in blood (neutrophils, monocytes, B cells), on macrophages and dendritic cells in tissues, and on podocytes in the kidney. In the brain, CR1 expression has been demonstrated in neurons and astrocytes in post-mortem LOAD and multiple sclerosis brain tissue [29,30,31]. CR1 expression has also been reported in cultured primary human astrocytes and microglia, and on human stem cell-derived microglia transplanted into mouse brain [31,32,33]; however, there is a continuing debate with some suggesting that CR1 is not expressed in the brain and that the impact of CR1 on AD is explained by its peripheral roles in IC handling [34]. A clear understanding of whether, where, and when CR1 is expressed in the brain is essential for our understanding of how *CR1* single nucleotide polymorphisms (SNPs) might confer increased LOAD risk.

### 5.3. Structure and Genetic Variants

**Figure 3:**
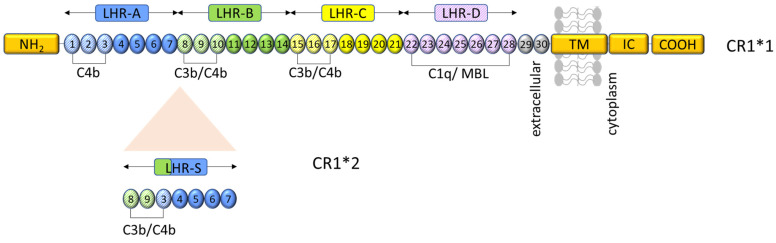
Representation of CR1 structure and ligand binding sites. CR1*1 comprises, from the amino terminus (NH_2_), four long homologous repeats (LHRs A-D), each composed of seven short consensus repeats (SCRs) of 60–70 amino acids each, two additional SCRs, a transmembrane segment (TM), and an intracytoplasmic carboxy-terminal domain (IC-COOH). Each circular block represents an SCR (numbered 1–30). There are three C4b binding sites (SCR 1–3, 8–10, and 15–17) and two C3b binding sites (SCR 8–10 and 15–17). SCRs 22–28 bind C1q, MBL, and ficolins. CR1*2 has an additional LHR domain (LHR-S) inserted between LHRs A and B and consequently an extra C3b/C4b binding site. Schematic based on similar figures in the work of [37,39,42].

The *CR1* gene is located on chromosome 1q32 within the regulators of complement activation (RCA) gene cluster; like other members of this cluster, it is a highly repetitive gene made up of repeating units with internal duplications that cause copy number variation (CNV). CNV in *CR1* generates four co-dominant alleles that encode CR1 proteins differing in the number of long homologous repeats (LHRs) (Figure 3). CR1*1 (also called CR1-A or CR1-F), a 190 kDa protein, is the most common variant with an allele frequency of 0.87; it comprises four LHRs, each made up of seven short consensus repeats (SCRs; 60–70 amino acid, internally disulphide-bonded structural units), an additional two membrane-proximal SCRs, transmembrane and cytoplasmic regions. CR1*2 (also called CR1-B or CR1-S) has an extra LHR, a duplication of SCR 3–9, yielding a 220 kDa protein; it has an allele frequency of 0.11. The remaining alleles, CR1*3 (also called CR1-C or CR1-F’; 160 kDa) and CR1*4 (also called CR1-D; 250 kDa), are very rare [35,36]. CR1*2 increases risk of LOAD by ~30% [3,9,30,37]. The addition of an extra LHR in CR1*2 increases the number of C3b/C4b binding sites, a theoretical gain of function (Figure 3) [38,39]. The increased risk associated with a gain-of-function variant in a molecule essential for IC clearance is counter-intuitive; one plausible explanation is that expression of the CR1*2 haplotype is reduced; indeed, reduced CR1 expression on erythrocytes in CR1*2 carriers has been reported [40,41]. It has been suggested that the expression of CR1*2 is reduced compared to CR1*1 because it is less efficiently trafficked to the membrane, remaining trapped in cytoplasmic vesicles [30]. Whether the CR1*2 allele is associated with a reduced expression on CNS resident immune cells remains to be demonstrated.

The most recent meta-analysis of LOAD GWAS identified rs679515 as the most significant *CR1* risk SNP [8]. Prior to this, rs4844610 and rs6656401 were reported [3,9]. All three SNPs are intronic, and all are in linkage disequilibrium. This SNP association marks the CNV described above, providing a means of identifying risk CNV carriers and clues to the mechanism [37,38]. A single rare coding variant, rs4844609, has been identified that is associated with episodic memory [43]. This SNP causes a Ser1610Thr substitution at a membrane-proximal site in LHR-D of CR1 previously implicated as a C1q binding site [44]. One study reported that the risk variant at this SNP increased the binding affinity of CR1 for C1q [31]; however, this was not replicated using recombinant CR1 LHR-D containing this Ser/Thr substitution [44]. Others suggested that the Ser1610Thr change altered susceptibility to enzymatic cleavage of CR1 and generation of soluble CR1 (sCR1), a locally active, fluid-phase complement inhibitor that might impact dysregulation of complement in the surrounding milieu. Indeed, increased plasma levels of sCR1 have been associated with both rs4844609 and rs6656401 [31,40]. It was suggested that rs4844609 accounts for the known LOAD risk effect of rs6656401 [43]; however, this has been refuted by others [45]. To date, the LOAD-associated SNPs in *CR1* were identified from GWAS in Caucasian populations [3,9,46,47,48]. The few analyses of non-European populations have reported conflicting results, some reports showing association of these same variants in *CR1* with LOAD in, for example, Han Chinese populations [49,50], whereas others failed to replicate the findings from Caucasian populations [51].

## 6. Clusterin

### 6.1. Function

Clusterin is a multifaceted protein; its many and diverse functions were discovered independently of each other; hence, clusterin has many names in the literature [52]. Clusterin is a lipoprotein that, in addition to roles in lipid transport, is an extracellular chaperone with roles in BAX-mediated apoptosis, PI3K pro-survival, and oxidative stress pathways [53,54,55,56]. Clusterin also contributes to the regulation of the complement system; it is a fluid-phase inhibitor of the terminal complement pathway, binding MAC precursors in the fluid phase to prevent membrane binding and pore formation [57,58].

### 6.2. Expression

Clusterin is ubiquitously expressed in tissues. Alternative splicing generates three forms of clusterin that are, respectively, nuclear, cytoplasmic, and secreted. The first two are regulators of apoptosis and intracellular chaperones and are not discussed further here. Secreted clusterin is present in plasma at a concentration of ~100 mg/L; a proportion of this will be contained within lipoprotein particles. Clusterin is also present in cerebrospinal fluid (CSF) and other biological fluids, notably at high levels in seminal plasma. Clusterin is abundantly expressed in the CNS, predominantly by astrocytes with region-specific expression in a subset of neurons [59,60]. In the healthy brain, astrocytes are responsible for the production and secretion of clusterin into the extracellular space. Overexpression of both neuronal and astrocytic clusterin has been reported in cases of inflammatory insult and neurodegenerative disease, including traumatic brain injury and spinal cord injury [61,62,63,64].

A role for clusterin in LOAD was first reported over 30 years ago. Clusterin mRNA is upregulated in AD tissue [65], and clusterin protein is abundant in the AD brain, where it is found in a subset of plaques and co-localises with MAC-labelled dystrophic neurites, neuropil threads, amyloid deposits, and intracellular neurofibrillary tangles [66,67]. Clusterin expression positively correlated with *ApoE4* allele number [68]. Levels of clusterin are elevated in the CSF and plasma of LOAD patients [69,70]; indeed, plasma clusterin has been suggested as a biomarker for AD, correlating with disease severity and progression from mild cognitive impairment (MCI) to AD in some studies [71,72,73]. Precisely how clusterin impacts the pathogenesis of LOAD remains unclear. In an in vitro acellular system, clusterin prevented Aβ aggregation [74]. Clusterin and the Clu-receptor glycoprotein 330/megalin have been reported to complex with soluble Aβ (sAβ) in the brain in order to facilitate the transport of sAβ across the blood-brain-barrier (BBB) [75]. Others have shown that clusterin binds and sequesters Aβ_1-40_ aggregates in vitro [76]. In mouse models, *Clu*^−/−^*ApoE^−/−^* double knockout mice showed markedly increased Aβ production and amyloid deposition compared with either single knockout, suggesting cooperative effects of these lipoproteins [77,78]. Recent studies have also suggested a role for clusterin at the synapse with increased clusterin protein reported in synaptoneurosomes from AD patients and in *ApoE4* carriers [79].

### 6.3. Structure and Genetic Variants

Clusterin is a heavily glycosylated heterodimeric protein comprising α and β chains each of ~40 kDa molecular weight, generated from an 80 kDa precursor protein and linked by five disulphide bonds. The structure is poorly defined, in part because of its tendency to aggregate; however, both chains contain stretches of amphipathic helix interspersed with disordered regions. The resultant molecule is highly flexible, likely explaining its broad range of binding partners. The gene encoding clusterin (*CLU*) is found on chromosome 8p21-12 and comprises nine exons. The primary transcript (NM_001831.3) encodes an immature pre-pro-protein containing a 22 amino acid signal sequence for translocation to the endoplasmic reticulum (ER). At the ER, immature clusterin is processed and cleaved to yield the highly glycosylated, mature heterodimeric protein.

Rare nonsynonymous mutations in *CLU* have been reported in a subset of AD patients and shown to result in intracellular accumulation of CLU in the ER and loss of secreted clusterin at the Golgi apparatus [80]. Of more relevance, there is an abundance of genetic evidence associating variants within the *CLU* gene with increased LOAD risk; indeed, *CLU* is the third strongest genetic risk factor for LOAD to date. Independent studies have identified multiple SNPs in *CLU,* associated with increased LOAD risk (rs11136000, rs2279590, rs9331888, rs9331896 and rs11787077) [8,9,26]. To date, there is no clear mechanism to explain how these clusterin variants confer increased LOAD risk, a task that is greatly complicated by the promiscuity of the protein. Whether and how SNPs in *CLU* affect clusterin synthesis systemically and locally in the CNS remains to be determined.

All SNPs studied to date have been suggested to affect plasma clusterin levels [81,82,83]. The rs11136000 SNP is located in intron 3 of *CLU*; 88% of Caucasians carry the C allele, this increases LOAD risk 1.6-fold [9]. The C allele is also associated with the risk of mild cognitive impairment (MCI) and progression from MCI to AD [83,84]. The minor T allele shows a mild protective effect [85]; this SNP has also recently been shown to be associated with cognitive decline in Parkinson’s disease patients [86]. The rs9331888 risk SNP has been associated with low levels of plasma clusterin and linked to alternative splicing of the *CLU* gene [82,87,88]. It should be stressed that *CLU* may impact LOAD risk independently of complement regulation via its roles in lipid handling and Aβ clearance; this has been expertly reviewed elsewhere [89].

## 7. C1S

### 7.1. Function

C1s is a single-chain glycoprotein, a highly specific serine protease, and a core component of the C1 complex, the initiator of the classical complement pathway. C1q is the recognition unit of the complex, binding antibody or other ligands; conformational changes in C1q then activate the associated pro-enzyme C1r, which in turn proteolytically activates pro-C1s. Activated C1s can then cleave C4 and C2 to form the C3 convertase C4b2a. Deficiency of C1s (or any of the components of the C1 complex) is strongly associated with a lupus-like immune complex disease reflecting loss of capacity to activate complement on immune complexes.

### 7.2. Expression

The *C1S* gene is located on chromosome 12, where the *C1R* and *C1S* genes lie end to end separated by 9.5kb; they are derived from a common ancestral gene through gene reduplication [90]. C1s are predominantly made in hepatocytes but are also produced by activated macrophages and monocytes. Brain expression is low and predominantly by microglia [91]. The plasma concentration of C1s is ~30 mg/L, the large bulk of this incorporated in the C1 complex. C1s are also present in CSF, although absolute levels were not obtained [92].

### 7.3. Genetic Variants

Complete C1s deficiency is associated with the immune complex disease as noted below; partial deficiencies have been associated with Ehlers-Danlos syndrome though the underlying mechanisms are unclear. Until very recently, no other disease-associated variants in *C1S* were reported. The most recent GWAS identified a novel SNP 5Kb upstream of *C1S,* which showed suggestive association with increased LOAD risk (SNP rs3919533) [8]. The mechanism of action of this SNP remains to be determined through functional experiments; however, given the location of the SNP, it is likely to impact the expression of the protein; indeed, C1s levels have previously been shown to be reduced in the CSF of AD patients, though there is no evidence that this observation is related to the *C1S* risk SNP [92].

## 8. Complement in LOAD: Smoking Gun or Red Herring?

In many chronic inflammatory and degenerative diseases, a role for complement has been clearly demonstrated, often with evidence pinpointing the relevant parts of the complement pathway involved in disease aetiology, for example, alternative pathway activation in AMD, and sometimes with proven efficacy of anti-complement drugs. Until very recently, the situation for LOAD was very different; complement proteins and activation products had been demonstrated in LOAD brains and biological fluids, but this “guilt-by-association” was not supported by solid evidence. Two things have changed the situation; first, the demonstration that complement activation at the synapse is a critical player in synapse loss in the disease; second, the genetic evidence implicating complement summarised above. The genetics tell us that *CR1*, *CLU,* and likely *C1S* are strongly implicated in the disease process—although whether the clusterin association involves its complement roles is very unclear. While this provides strong evidence that complement dysregulation is involved in LOAD, it does not point towards a specific pathway or mechanism.

Understanding how complement variants confer LOAD risk is further complicated by several factors. Firstly, the majority of the LOAD-associated complement variants identified to date are non-coding and likely confer risk by affecting cell and region-specific expression levels. Unlike in other more accessible organs, the location and nature of the brain make it impossible to assess longitudinal expression levels in the brain parenchyma, and reliance on post-mortem evidence likely masks important early and progressive changes. Second, LOAD-associated complement genes predispose individuals to LOAD, but other risk factors (non-complement and non-genetic) are required to cause disease; hence, functional studies of risk variants must be conducted in specific contexts to reveal relevant mechanistic pathways of action. In vivo and in vitro studies, each with different limitations must be used in conjunction to understand the role of complement at different time points in disease. Third, there are many regions of the human genome, including some important complement loci, which cannot be assembled or aligned using standard short-read sequencing technologies, preventing the identification of disease-causing mutations or variations [93,94]. These regions are referred to as “dark” or “camouflaged”; “dark” regions are difficult to sequence due to, for example, high GC content, while “camouflaged” regions of the genome are highly repetitive, making alignment of short reads difficult. The complexity of complement genes is a consequence of gene reduplication events, so many loci, notably the RCA cluster, are highly repetitive in nature and therefore likely well camouflaged. For example, regarding *CR1* in the RCA cluster, 26% of the protein-coding region is hidden due to its highly repetitive nature so that significant variation may be missed by standard sequencing in GWAS [94]. Indeed, this study, systematically targeting “dark” genes relevant to LOAD risk, identified a novel 10-nucleotide frameshift mutation in CR1 present in five cases but no controls.

Our recent study using available GWAS data identified no remaining complement gene LOAD association when *CLU* and *CR1* were removed from a complement geneset [95]; however, such analyses are limited by the data. Indeed, the recent GWAS identification of a suggestive association of *C1S* with LOAD [8] highlights that larger data sets and newer sequencing technologies may identify other complement genes that impact LOAD risk.

Often, by the time people with LOAD reach the clinic, they already have significant irreversible pathology. Understanding of complement risk genes and the resultant complotypes involved in LOAD might facilitate predictive genetic screening tests; if the complotypes can be linked with complement levels in plasma, as seen in AMD, this might enable the development of simple diagnostic tools and guide the future use of anti-complement drugs in LOAD. There are a number of anti-complement therapeutics currently in development for CNS disorders [96]; genetic and biomarker assays could be used to stratify patients for anti-complement therapeutic interventions.

## Figures and Tables

**Table 1:** Complement gene variants and associated diseases.

Gene	Variant	Disease
C1q	Deficiency Polymorphism	Increased risk of lupus and glomerulonephritis Arthritis, cancer, diabetes, schizophrenia
C1r/C1s	Deficiency GOF SNP	Autoimmunity, infections, glomerulonephritis, Type I periodontal Ehlers-Danlos Increased risk of AD
C1INH	Deficiency	Hereditary angioedema (types I and II)
C2	Deficiency SNPs	Lupus, bacterial infections Protective for AMD and PCVP
C3	GOF Nonsynonymous Coding variant	aHUS, C3G, and AMD
C4	Deficiency CNV	Lupus Schizophrenia
C5	Nonsense; hom or Compound het	C5 deficiency; neisserial infections
C6	Single bp deletion	C6 deficiency; neisserial infections
C7	Nonsense: hom or compound het	C7 deficiency; neisserial infections
C8α	Nonsense: hom or compound het	C8 deficiency, type I; neisserial infections; no C8α protein; free C8β
C8β	Premature stop codon	C8 deficiency, type II; neisserial infections; no C8β protein; free C8α
C9	Nonsense: hom or compound het SNPs	C9 deficiency; neisserial infections AMD; AD
MASP-1, collectins	Hom/het deficiency	Various developmental; Malpuech, Carnevale, Michels, and Mingarelli syndrome
Ficolins	SNPs	Rheumatoid arthritis, leprosy, systemic inflammation, bacterial infections
CFH	Hom deficiency SNPs and truncations	DDD; MPGN C3G; acquired partial lipodystrophy; aHUS AMD; AD; Some protective against meningococcal disease, AMD, IgAN, or C3G
CFI	Nonsense: hom, het or compound het	AMD; C3G; aHUS; recurrent infections
MCP	Hom/Het deletion/truncation Missense SNP	Systemic sclerosis, miscarriage, HELLP syndrome, and C3G Severe aHUS; linked to CVID
CFB	Nonsense: hom or compound het Het GOF SNP Other SNPs	Factor B deficiency; recurrent bacterial infections aHUS Protection against AMD
Properdin	Nonsense/truncating mutations	Properdin deficiency (X-linked); neisserial infections
DAF	Nonsense: hom or compound het	CHAPLE Syndrome; linked to Inab Cromer blood group
CD59	Nonsense: hom or compound het	CD59 deficiency; PNH-like disease; Peripheral neuropathy; strokes
CFHR1/3	Combined gene deletion	Risk for aHUS; protection from AMD
CFHR5	Gene duplication SNPs	aHUS C3G; poststreptococcal glomerulonephritis
Clu	SNPs	AD
CR1	SNPs	AD

AD—Alzheimer’s disease, aHUS—atypical haemolytic uremic syndrome, AMD—age-related macular degeneration, bp—basepair, C3G—complement 3 glomerulopathy, CHAPLE—complement hyperactivity, angiopathic thrombosis, and protein-losing enteropathy, CNV—copy number variant, CVID—common variable immunodeficiency, DDD—dense deposit disease, GOF—gain of function, het—heterozygous, hom—homozygous, MPGN—membranoproliferative glomerulonephritis, LOF—loss of function, PCVP—polypoidal choroidal vasculopathy, PNH—paroxysmal nocturnal hemoglobinuria, SNP—single nucleotide polymorphism.
